# The role of dietary sugars in health: molecular composition or just calories?

**DOI:** 10.1038/s41430-019-0407-z

**Published:** 2019-02-20

**Authors:** Philip Prinz

**Affiliations:** Department of Nutritional Sciences, German Sugar Association, Berlin, Germany

**Keywords:** Obesity, Metabolic diseases, Metabolic disorders, Obesity, Metabolic diseases

## Abstract

This review will focus on the question of whether dietary sugars are a relevant determinant in the global rise of overweight and obesity in adults, adolescents, and children. Initially, the review describes the current definitions for sugars in the diet and makes reference to them while analyzing their role in overweight and obesity as well as diet-related diseases, including type 2 diabetes, cardiovascular diseases, non-alcoholic fatty liver disease and cancer. Second, it will focus particularly on sucrose and the question of whether it is the molecular composition of sucrose (glucose and fructose) or its energy content that promotes body weight gain and diet-related diseases. Finally, the review will clarify the molecular characteristics of sucrose regarding the release of the gastrointestinal glucose-dependent insulinotropic peptide (GIP) compared to other energy-providing nutrients and its relevance in metabolic diseases. Current data indicates that dietary sugars are only associated with an increase in obesity when consumed as an excess source of calories and with that an increase in the risk of diet-related diseases. Furthermore, it was shown that a diet rich in fat will stimulate GIP secretion more than a diet rich in sucrose. Taken together, current scientific evidence does not support the conclusion that dietary sugars per se are detrimental to human health.

## Introduction

In Central Europe, average body weight has constantly increased in adults, adolescents and children during the last four decades [1], a finding that is reflected by the global rise of overweight and obesity [2]. Since 1980, obesity has doubled in more than 70 countries and is a major risk factor for several noncommunicable diseases (NCDs) [3].

For years, there has been a public debate about dietary sugar intake and its role in the development of obesity and NCDs, including type 2 diabetes (T2D), cardiovascular diseases (CVDs), and cancer. There are several definitions of dietary sugars available; they include “added sugars”, which are all sugars and syrups that are added to foods during processing and preparation, “free sugars” which according to the definition of the World Health Organization (WHO) comprise all mono- and disaccharides that are added to foods by the manufacturer, cook or the consumer as well as the sugars that are naturally present in honey, syrups, and fruit juices. The definition “total sugars” includes all sugars that naturally occur in food as well as added sugars [4]. These definitions naturally include all monosaccharides (e.g., glucose, fructose, and galactose) and all disaccharides (e.g., sucrose, lactose, and maltose) [4]. Since these definitions are often mixed up, the review will refer exactly to the definitions used in the original works to avoid confusion. A detailed description of the different types of sugars can be found elsewhere [4].

The review will highlight current literature to discuss the question of whether dietary sugars per se have an unfavorable health effect or if it is just the amount of calories that matters in the development of NCDs and obesity.

## The role of sugar in body weight and noncommunicable diseases

### Overweight and obesity

Obesity is defined by a body mass index (BMI) >30 kg/m² and it affects the quality of life as well as the lifespan remarkably [5, 6]. Although the development of obesity is multifactorial, the main reason is always a positive energy balance [7]. Such positive energy balance results from an increased intake of energy from foods or beverages (calories), which exceeds the energy expenditure (including basal metabolism, thermogenesis, or physical activity) [7, 8]. Finally, a prolonged positive energy balance will result in overweight and obesity. Besides energy balance, it is only biological factors, including sex, age, genetics, and hormones that have a direct impact on the development of overweight and obesity [8]. Other factors, including socioeconomic status, lifestyle, or mental factors have only an indirect effect on overweight and obesity [8].

The role of dietary sugars in the development of obesity has been investigated in various meta-analyses, which deliver the highest quality of scientific evidence. Te Morenga and colleagues on behalf of the WHO investigated the role of free sugars at the onset of obesity by performing a systematic review and meta-analyses including randomized controlled trials as well as prospective cohort studies, which concluded that free sugars are a crucial determinant for body weight gain. A high intake of free sugars is associated with an excess calorie intake, which if not compensated by energy expenditure will lead to an increase of body fatness [9]. Since the isocaloric exchange of free sugars with other carbohydrates was not associated with weight changes, the authors concluded that this effect is mediated by changes in energy intake [9]. These results were confirmed by subsequent analyses of independent researchers. Fattore et al. performed a systematic review and meta-analyses in 2017 that showed that free sugars had no effect on body weight as long as they were isocalorically exchanged with other carbohydrates [10]. Therefore, through the highest scientific evidence of human studies, it can be concluded that the consumption of free sugars will only result in overweight and obesity if more calories in the form of free sugars are consumed than expended. Free sugars per se do not favor body weight gain as shown by isocaloric exchange with other carbohydrates.

It should be kept in mind that increased energy consumption is not only mediated by the intake of sugars but can also be promoted by foods with a high energy density [11]. The energy of foods is mainly determined by its water (0 kJ/g) and fat (37.7 kJ/g = 9 kcal/g) content. Therefore, foods with a high water content have a low energy density and foods with a high content of fat have a high energy density. Additionally, foods which are rich in fiber (9.6 kJ/g = 2.3 kcal/g) also have a low energy density; this includes fruits and vegetables [11]. For body weight management, it is indispensable to refer to the energy density of foods [11]. In order to tackle overweight and obesity, reducing sugars in foods will only be successful if the intake of calories via sugars (16.7 kJ/g = 4 kcal/g) is also reduced and not substituted with other energy-providing nutrients. To lose body weight, total energy intake needs to be reduced.

In beverages, sucrose can be replaced by artificial sweeteners, resulting in reduced energy content, whereas in solid foods, it is very difficult to reduce the amount of sucrose without changing technical properties and taste. Besides its sweetness, sucrose is important for bulk and texture as well as flavor formation of, e.g., bakery products. It reduces the water activity in foods resulting in increased shelf-life and is used for yeast fermentation [12, 13]. Currently, there is no other sweetener that can duplicate all or many of the functional properties of sucrose [13]. Technical research has indicated that sucrose cannot simply be replaced by a single nutrient, but a mixture of nutrients or compounds must be used to replace the functional attitudes and sweetness of sucrose [12]. Taken together, sucrose cannot easily be replaced by other nutrients in solid foods to reduce calorie content without affecting functional properties. If sucrose is replaced by other carbohydrates, the taste will diminish while the caloric value will not change; however, the replacement may have a different effect on blood glucose levels [14]. If replaced by fat, it can be assumed that the calorie content of solid foods will increase.

Apart from solid foods, the role of sugar-sweetened beverages (SSB) in the development of overweight and obesity is continually being discussed. The consumption of beverages has a lesser feeling of fullness with a faster recurring feeling of hunger, resulting in an increased energy intake compared to the group that received energy matched solid foods [15]. The results were confirmed by other studies that compared the effect of fruits and vegetables provided in either solid or liquid form [16]. Altogether, the problem with energy-containing beverages is that, even with the same amount of calories compared to solid food, they are less filling. Thus, there is the risk of an increased intake of calories in the subsequent meal.

Besides their less pronounced effect on satiety, SSBs are always discussed to induce weight gain due to their amount of added sugars. Systematic reviews including meta-analysis of prospective cohort studies show that an increased intake of SSBs is associated with an increase of body weight in children and adults [9, 17]. Since observational studies cannot show causality, results from intervention trials are needed. Interestingly, a systematic review and meta-analysis of intervention trials in children that were advised to reduce consumption of SSBs and other dietary sugars did not result in reduced body weight, but most of the studies that were investigated showed poor compliance for dietary advice [9]. Additionally, the authors showed that free sugars (including those from SSBs) did not lead to weight gain, if there was isocaloric exchange with other carbohydrates [9]. However, independent meta-analysis showed that replacement of SSB in the diet with noncaloric beverages resulted in decreased body weight [17]. For adults, exclusively addition studies were reviewed, which showed that SSB consumption in addition to the normal diet increased body weight [17].

All in all, beverages are less satiable than solid foods that contain the same amount of calories, which can result in an increased caloric intake of the subsequent meal. When body weight gain should be avoided via calorie reduction, SSBs must be assessed in the same way as other energy-containing beverages. To reduce body weight, SSBs should not be consumed in addition to the normal diet as they provide excess calories, whereas a consumption under isocaloric conditions seems not to affect body weight [9].

### Type 2 diabetes (T2D)

Most of the available data on the effect of sugars in the development of T2D is available from observational studies. Since T2D is a disease that develops over years, it is not possible to investigate the impact of sugars on the long-term development of T2D in intervention studies. In 2010, the European Food Safety Agency (EFSA) stated that the available scientific data is insufficient for setting an upper level for the intake of added sugars based on their effect on T2D [18]. Subsequently, Hauner and colleagues investigated the role of various carbohydrates on behalf of the German Nutrition Society (DGE). In line with the results of EFSA, they concluded that the association between the total intake of all mono- and disaccharides, including glucose and fructose, and the risk of T2D is considered to be insufficient. Added to that and because of the inconsistency of the results, the evidence regarding the lack of an association between sucrose intake and the risk of T2D is viewed as probable [19]. In 2015, the British Scientific Advisory Committee on Nutrition (SACN) also stated that there is no association of daily intake of all dietary sugars and T2D due to limited evidence [20]. In line with these results, a meta-analysis of prospective cohort studies concluded that total sugar and fructose cannot be associated with the onset of T2D and interestingly sucrose was associated with a decreased risk of T2D [21]. All in all, current data does not support the theory that dietary sugars support the development of T2D. Therefore, the assumption that dietary sugars alone will cause T2D is scientifically not evaluated and wrong; however, obesity as a result of an unhealthy lifestyle is strongly related with the incidence of T2D [22].

As regards to sucrose, there is an old paradigm indicating that sucrose has a high glycemic index (GI) that leads to a fast increase in blood glucose followed by a rapid decrease, which then initiates a new feeling of hunger. The GI is defined by the rise in the blood glucose level after ingestion of carbohydrates compared to glucose (=100) and shown as an area under the curve (AUC) [23]. A systematic review of 15 prospective cohort studies showed that a high GI is associated with an increased risk of T2D [24]. Interestingly, according to Atkinson and colleagues a high GI is defined as being 70 or greater and is related to breads, breakfast cereals or rice, whereas a low GI is 55 or less and is related to legumes, pasta, fruits, and dairy products [14]. Sucrose has a medium GI of 65 [14]. This shows that other foods such as breads or cereals, which are made of processed grains, may have a higher impact on blood glucose levels than sucrose. Alternatively, not all processed foods have a high GI in general, there are also processed foods with a medium GI comparable to that of sucrose, including various chocolate or cereal bars, cakes or certain breakfast cereals [14]. In this case, replacing sucrose with processed grains or other sources of carbohydrates may result in a product with a higher GI and thus a greater impact on the glycemic response. This is a relevant aspect regarding current efforts to reformulate foods and to reduce the amount of sucrose.

Particularly SSB consumption and its role in the development of T2D promote controversy when discussed. A systematic review and meta-analysis of prospective cohort studies by Malik and colleagues indicated that a higher consumption of SSBs (1–2 servings per day) increases the risk of developing T2D by 26% compared to a lower consumption of SSBs (one serving per day or less) [25]. The authors also stated, that body weight gain partly contributes to the development of T2D [25]. Because of the observational character of the studies (which cannot show causality) included in this systematic review and meta-analysis, it is possible that high SSB consumption contributes to the development of T2D by providing extra calories to the normal diet.

Another systematic review and meta-analysis of prospective cohort studies showed that high SSB consumption is positively associated with the risk of T2D independently of obesity. However, this finding was also described for the consumption of fruit juice and artificially sweetened beverages, respectively [26]. Since artificially sweetened beverages have no calories or a very low-calorie content, it seems to be likely that besides weight gain due to extra calorie intake, additional confounding factors contribute to T2D. These confounding factors can include lifestyle-factors, such as smoking, physical activity, alcohol and coffee consumption or consumption of red meat as well as other factors, including the socioeconomic status or existing hypertension [26, 27]. Although several of the potential confounding factors were excluded by most of the prospective cohort studies investigated in the systematic review and meta-analysis of Imamura and colleagues [26], generally not all of them can be excluded to generate causal hypotheses. Additionally, reverse causality is an important factor when discussing the outcome of observational studies. Reverse causality means that a disease might influence the dietary exposure rather than vice versa. Based on the systematic review and meta-analysis of Imamura and colleagues, it can be stated that the diagnosis of T2D might change the patient’s approach to health consciousness and with that the habitual consumption of beverages, which might result in an increased intake of artificially sweetened beverages. These results show that further research is necessary to investigate the role of SSBs, fruit juices and artificially sweetened beverage in the development of T2D. Since several confounding factors cannot be excluded by prospective cohort studies, it cannot be clearly stated that (isolated) SSB consumption leads to the development of T2D.

Interestingly, a systematic review and meta-analysis of Xi and colleagues concluded that the increased intake of sugar-sweetened fruit juice was associated with the incidence of T2D, whereas an intake of 100% fruit juice was not [28]. It is noteworthy, that in the previous systematic review and meta-analysis by Imamura and colleagues, ‘SSBs’ were defined as any sweetened beverages, including sugar-sweetened fruit juice, whereas ‘fruit juice’ was defined as 100% fruit juice, or fruit juice assessed separately from fruit drinks [26]. Since SSBs and fruit juices were associated with an increased risk for T2D, the authors concluded that fruit juices (including 100% fruit juice) cannot be seen differentially from SSBs [26]. These results are supported by independent researchers, pointing out that fruit juice consumption is not substantially different regarding health risks from consumption of SSB, because fruit juices have a similar energy density and sugar content compared with SSBs [29]. Taken together, the results of both systematic review and meta-analysis are controversial and need further research regarding the effect of SSBs as well as fruit juices (including 100% fruit juice) on T2D.

Interestingly, a recently published meta-analyses of controlled intervention trials showed that SSBs do not have a detrimental effect on glycemic control in energy matched substitution studies when sugars are substituted for other macronutrients (including HbA1c as well as fasting blood glucose and insulin) [30]. It is only when adding excess calories to the diet that SSBs have a harmful effect on glycemic control [30], pointing to a positive energy balance rather than dietary sugars per se as a crucial factor in the development of T2D.

All in all, the results from prospective cohort studies and controlled intervention trials indicate that SSBs contribute to the development of T2D by adding excessive calories to the diet resulting in a positive energy balance, but no effects on parameters of glycemic control were seen under isocaloric conditions [30]. All energy-containing beverages, including SSBs as well as fruit juices, should be seen in the same way in their effects on health due to their similar amount of calories and sugar content.

### Cardiovascular diseases (CVDs)

Most of the observational studies indicate a direct association of dietary sugars and the development of CVDs as demonstrated in a very large prospective cohort study from the United States with over 30,000 participants, which showed that a higher added sugar consumption is associated with increasing risk of CVD mortality in adults [31]. These studies can indicate correlation, but they cannot demonstrate causality due to various confounding factors that cannot be excluded, because of the observational character of these studies. To demonstrate causality, intervention trials are mandatory for scientific and political recommendations. There are currently two systematic reviews and meta-analyses of intervention trials available that investigated the role of dietary sugars in the development of CVDs. In 2014, Te Morenga and colleagues concluded that free sugars have a moderate effect on blood lipids and blood pressure, irrespective of their energy content when there was isocaloric exchange with other carbohydrates [32]. The results were very heterogenous and hard to interpret, since low- as well as high-density lipoproteins were increased through the isocaloric exchange of carbohydrates by free sugars. Added to that, the systolic blood pressure decreased, whereas the diastolic blood pressure increased with the isocaloric exchange. Under conditions of ad libitum, free sugars increased blood lipids and blood pressure, pointing to a result of excess calorie intake [32]. Isocaloric exchange of free sugars with other carbohydrates did not point to a clear direction in the development of CVDs and needed further research. In 2017, Fattore and colleagues performed a subsequent systematic review and meta-analysis that focused on free sugars and their effect on CVDs. The authors concluded that exchanging ‘complex carbohydrates’ with free sugars does not affect blood lipids or blood pressure [10]. These results indicate that free sugars per se have no effect on blood pressure and blood lipids. Unfortunately, Fattore and colleagues did not investigate the effects on CVDs when free sugars where consumed as excess energy [10]. To further support the hypothesis that dietary sugars per se do not increase the risk of CVDs, it was concluded that fructose does not affect blood lipids in isocaloric controlled feeding trials [33]. Only fructose intake, additional to the existing diet, providing excess energy intake resulted in increased blood lipids [33]. Regarding fructose, these findings are further supported by another systematic review and meta-analysis, showing that isocaloric exchange of fructose for other carbohydrates in clinical trials decreased diastolic blood pressure and mean arterial blood pressure without affecting systolic blood pressure [34]. Additionally, hypercaloric fructose intake did not affect overall mean arterial blood pressure in feeding trials compared with other carbohydrates [34].

All in all, these findings are in line with analysis from international nutritional organizations, which concluded that added sugars as well as other dietary sugars are not associated with CVDs [19, 20] and that the current data is insufficient to set an upper level [18]. Current scientific evidence from human intervention studies indicates that dietary sugars per se do not cause CVDs, but high sugar consumption, which exceeds energy expenditure, can cause body weight gain and obesity due to excessive calorie intake [9]. It is well known that obesity is a major risk factor for CVDs [35] and therefore, nutritional recommendations to prevent CVDs should focus on tackling obesity and reducing calorie intake rather than focusing on the reduced intake of a single nutrient.

### Cancer

Around the 1920s, Otto Warburg and colleagues described that cancer cells favor anaerobic glycolysis to generate energy for cellular processes rather than normal cells, which rely primarily on mitochondrial oxidative phosphorylation, a phenomenon termed the “Warburg effect” [36]. This discovery lead to the assumption that decreasing glucose intake due to reduced intake of dietary sugars (e.g., ketogenic diets) leads to a glucose ‘starvation’ of tumor cells and also potentially reduces insulin-related cell growth as a result of the reduction of blood glucose levels [37]. Today, discussions continually recur as to whether dietary sugars are involved in different types of cancer.

Recently, Makarem and colleagues performed a systematic review of 37 prospective cohort studies on the role of dietary sugars in cancer risk [38]. The results indicate that most of the available literature reported a null association of total sugar and sucrose intake to cancer risk, respectively. Of 14 studies on fructose, eight reported a null association, two a protective and four a detrimental association to cancer risk. Contrary, a higher intake of added sugars and SSBs was observed to be associated with a higher cancer risk in half of the studies that were analyzed. The authors concluded that most studies indicated that dietary sugars and cancer risk have a null association but added sugars and SSBs might be detrimental when associated with cancer risk [38]. Since these findings are made from observational studies, the results must be viewed with caution. Hauner and colleagues evaluated current literature regarding the association between mono- and disaccharides and the risk of cancer. Except for possible evidence of an association of monosaccharides intake and pancreatic cancer, there are no associations of mono- or disaccharides with different types of cancer due to a lack in scientific evidence or more importantly insufficient evidence [19]. These findings are supported by the current report of the World Cancer Research Fund (WCRF), which does not even refer to dietary sugars as a relevant nutritional parameter in the development of cancer [39]. Interestingly, the WCRF reported that there is strong evidence between increased body fat and the risk of cancer. Therefore, the WCRF recommends keeping a healthy body weight balance by increasing physical activity and reducing the amount of fast food [39]. These recommendations clearly point to a healthier lifestyle in order to reduce the risk of cancer by reducing body weight. The role of body weight in the development of cancer is further supported by a systematic review and meta-analysis, which showed that an increase in BMI by 5 kg/m² increases the risk of various types of cancer in men and women [40]. Overall, there is very little evidence that dietary sugars are associated with different types of cancer. To reduce the risk of cancer, a healthy lifestyle with a moderate body weight is essential.

## Molecular structure of sucrose and its role in energy metabolism

Recently a study by Pfeiffer and colleagues concluded that sucrose due to its specific structure (a disaccharide of glucose and fructose bound by an α-1,2-glycoisidic linkage) and independent of its energy content leads to increased secretion of the gastrointestinal glucose-dependent insulinotropic peptide (GIP). More specifically, glucose is responsible for the increased GIP secretion, which in turn increases appetite, body weight and the risk for insulin resistance. Fructose as the second component of sucrose promotes the development of the non-alcoholic fatty liver disease (NAFLD) [41]. The results of this study were correct as far as the GIP level increased after oral administration of sucrose to mice and humans compared to isomaltulose. Isomaltulose also consists of glucose and fructose, but it has an α-1,6-glycosidic bond, which allows slower enzymatic digestion in the small intestine and consequently decreased secretion of GIP and a slower increase of blood glucose and insulin levels [41], suggesting that its usage in, e.g., SSBs could be beneficial for energy metabolism. But it has to be kept in mind that isomaltulose has only half of the sweetness of sucrose [42], which might result in higher amounts in SSBs to maintain sweetness and with that a resulting different GIP response (and a higher amount of calories).

However, the main problem of this study was that it is not transferable to the human diet because sucrose or isomaltulose are rarely eaten individually. To understand the effect of sucrose on GIP secretion, the whole diet must be taken into consideration. Raben and colleagues investigated the effect of a high-fat diet (46% fat) with a high-starch (59% carbohydrates, of which 2% sucrose) and a high-sucrose diet (59% carbohydrates, of which 26% sucrose) on the secretion of gastrointestinal peptides in women over 15 days under ad libitum conditions [43]. Postprandial blood tests on day 15 showed significantly increased GIP secretion in the group with the high-fat diet compared to the high-starch and high-sucrose diet. No differences were found for the high-starch and high-sucrose diet. Although all participants received an ad libitum diet, total energy intake of the group receiving the high-fat diet and the high-sucrose diet were not different during the 2 weeks, and the day of blood withdrawal. Only the group receiving the high-starch diet had a significantly reduced total energy intake during the 2 weeks, and the day of blood withdrawal compared to the high-fat and high-sucrose-diet, respectively [43]. In line with these findings, a crossover trial investigated the effect of either 500 ml regular cola (sucrose-sweetened, 900 kJ), semi-skimmed milk (950 kJ), diet cola (7.5 kJ), or water on appetite scores and secretion of gastrointestinal peptides, including GIP [44]. The results showed that semi-skimmed milk significantly increased GIP secretion compared to isocaloric regular cola [44], indicating that fat is a more potent stimulator of GIP than sucrose.

All in all, these results demonstrate that a diet rich in fats or a beverage that contains fat will stimulate GIP secretion much more than a diet rich in sucrose or a SSB. These findings disagree with the hypothesis that sucrose because of its molecular structure has a detrimental effect as far as the release of GIP and the support of body weight gain and insulin resistance is concerned. Apart from GIP, several other gastrointestinal peptides are involved in the regulation of appetite, glucose homeostasis and thereby affecting body weight. This includes cholecystokinin (CCK), peptide tyrosine tyrosine (PYY) glucagon-like peptide 1 (GLP-1), gastrin, secretin, or ghrelin. All of these peptides differ in the way they are released by nutrients, postprandial feedback mechanisms and by that, in the particular functions they have on physiology [45–47]. Therefore, when focusing on the sucrose-mediated release of gastrointestinal peptides, it is highly unlikely that solely GIP will have an impact on insulin secretion and blood glucose levels. Additional research is needed to further show the effects of sucrose alone on gastrointestinal peptides and postprandial physiology.

The second hypothesis and also a public opinion is that fructose directly induces NAFLD. This theory is only correct with regard to excess calorie consumption, as was demonstrated in a systematic review and meta-analysis of Chiu and colleagues [48]. The isocaloric exchange of fructose with other carbohydrates did not raise the amounts of intrahepatocellular lipids and alanine aminotransferase, both of which are markers for NAFLD. However, excess energy ingested via extreme doses of fructose did raise intrahepatocellular lipids and alanine aminotransferase [48]. The authors concluded that this effect might be more attributable to excess energy intake than to fructose [48]. These results are in line with findings, which demonstrated that fructose does not increase body weight per se but only if excess intake exceeds energy expenditure [49]. This suggests that an excess of calories from fructose rather than fructose itself is detrimental to human health.

In contrast, there are other results that potentially demonstrate that an isocaloric exchange of fructose with starch (the amount of fructose was reduced from 12 to 4%) in obese children significantly reduced the hepatic amount of lipids during 9 days [50]. Interestingly, the children also significantly lost weight during the 9 days of intervention [50]. Added to that the data from the children was used in a previous study of Lustig and colleagues; they described that 33 of 43 participants were not able to consume all of the isocaloric food provided to maintain body weight [51]. Therefore, the results of both studies are questionable, because most of the children did not eat the isocaloric amount of food and thus significantly lost body weight [50, 51]. This can be appropriate for reducing liver fat, as it has already been demonstrated that weight loss itself is appropriate to improve NAFLD [52].

## Conclusion

Current scientific evidence does not support the conclusion that dietary sugars themselves are detrimental to human health and the cause of obesity as well as NCDs. Data from human studies clearly shows that it is the excess amount of calories, also consumed in form of dietary sugars, that promotes obesity and with that favors NCDs. For sucrose, further research is needed in order to evaluate the relevance of its molecular composition, especially in comparison with other macronutrients.
